# Canadian Consensus Recommendations for Predictive Biomarker Testing in Gastric and Gastroesophageal Junction Adenocarcinoma

**DOI:** 10.3390/curroncol31120572

**Published:** 2024-12-04

**Authors:** Christine Brezden-Masley, Pierre O. Fiset, Carol C. Cheung, Thomas Arnason, Justin Bateman, Martin Borduas, Gertruda Evaristo, Diana N. Ionescu, Howard J. Lim, Brandon S. Sheffield, Sara V. Soldera, Catherine J. Streutker

**Affiliations:** 1 Temerty Faculty of Medicine, University of Toronto, Toronto, ON M5S 1A8, Canada; christine.brezden@sinaihealth.ca; 2 Medical Oncology, Mount Sinai Hospital, Toronto, ON M5G 1X5, Canada; 3 Department of Pathology, McGill University Health Centre, Montreal, QC H4A 3J1, Canada; pierre.o.fiset@mcgill.ca (P.O.F.); gertruda.evaristo@mcgill.ca (G.E.); 4 Laboratory Medicine Program, University Health Network, Toronto, ON M5G 2C4, Canada; carol.cheung@cbqa.ca; 5 Department of Laboratory Medicine and Pathobiology, University of Toronto, Toronto, ON M5S 1A8, Canada; 6 Queen Elizabeth II Health Sciences Centre, Halifax, NS B3H 3A7, Canada; thomas.arnason@nshealth.ca; 7 Department of Pathology, Dalhousie University, Halifax, NS B3H 4R2, Canada; 8 Alberta Precision Laboratories, Edmonton, AB T5H 3V9, Canada; justin.bateman@albertaprecisionlabs.ca; 9 Department of Pathology, Centre Hospitalier Universitaire de Sherbrooke (CHUS), University of Sherbrooke, Sherbrooke, QC J1H 5H3, Canada; martin.borduas@usherbrooke.ca; 10 BC Cancer, Vancouver, BC V5Z 4E6, Canada; dionescu@bccancer.bc.ca (D.N.I.); hlim@bccancer.bc.ca (H.J.L.); 11 Department of Pathology and Laboratory Medicine, University of British Columbia, Vancouver, BC V6T 2B5, Canada; 12 Division of Advanced Diagnostics, William Osler Health System, Brampton, ON L6R 3J7, Canada; brandon.sheffield@williamoslerhs.ca; 13 Division of Medical Oncology, Royal Victoria Hospital, McGill University Health Centre, Montreal, QC H4A 3J1, Canada; sara.soldera@mcgill.ca; 14 Unity Health Toronto, University of Toronto, Toronto, ON M5B 1W8, Canada

**Keywords:** gastric cancer, immunohistochemistry, IHC, HER2, MMR, CLDN18.2, CLDN18, PD-L1, immunotherapy, targeted therapy

## Abstract

Gastric cancer is common globally and has a generally poor prognosis with a low 5-year survival rate. Targeted therapies and immunotherapies have improved the treatment landscape, providing more options for efficacious treatment. The use of these therapies requires predictive biomarker testing to identify patients who can benefit from their use. New therapies on the horizon, such as CLDN18.2 monoclonal antibody therapy, require laboratories to implement new biomarker tests. A multidisciplinary pan-Canadian expert working group was convened to develop guidance for pathologists and oncologists on the implementation of CLDN18.2 IHC testing for gastric and gastroesophageal junction (G/GEJ) adenocarcinoma in Canada, as well as general recommendations to optimize predictive biomarker testing in G/GEJ adenocarcinoma. The expert working group recommendations highlight the importance of reflex testing for HER2, MMR and/or MSI, CLDN18, and PD-L1 in all patients at first diagnosis of G/GEJ adenocarcinoma. Testing for NTRK fusions may also be included in reflex testing or requested by the treating clinician when third-line therapy is being considered. The expert working group also made recommendations for pre-analytic, analytic, and post-analytic considerations for predictive biomarker testing in G/GEJ adenocarcinoma. Implementation of these recommendations will provide medical oncologists with accurate, timely biomarker results to use for treatment decision-making.

## 1. Introduction

Gastric cancer (GC) ranks as the fifth most prevalent cancer globally, with a higher incidence among men compared to women [1]. According to the Canadian Cancer Society, around 4000 Canadians are projected to receive a diagnosis of GC in 2024 [2]. Over the last five decades, there has been a consistent decline in the global occurrence of GC, attributed to advancements in preventing and treating *Helicobacter pylori* infection, which is a leading cause of GC, improvements in food preservation methods, and shifts in dietary habits [3]. Although the incidence of GC in Canada is declining, there is a steady increase in gastroesophageal junction (GEJ) cancer, and the prognosis is still generally poor, with a projected 5-year net survival rate in Canada below 30% [2]. This is because most cases are diagnosed at an advanced stage of disease since there are typically few symptoms at earlier stages, and there is no routine screening [4,5].

The most common type of GC is adenocarcinoma, which accounts for 95% of cases. GC can arise in the cardia/proximal stomach or distally in the antrum or pylorus [5]. Adjacent to the proximal cardia, adenocarcinomas occurring in the gastroesophageal junction are treated similarly to gastric adenocarcinomas in the advanced setting [6]. In the stomach, these adenocarcinomas are predominantly the intestinal or diffuse subtypes (WHO tubular or poorly cohesive carcinomas). At the GEJ, diffuse-type carcinomas are rare; the majority are intestinal-type adenocarcinomas [7,8,9].

The paucity of specific data on GEJ cancers in Canada highlights the need for dedicated databases and prospective studies to better understand the incidence, prevalence, and outcomes of these cancers within the Canadian population [10]. However, esophageal adenocarcinoma, including cancer of the distal GEJ, has shown a rising incidence not only in Canada but also in many other populations globally. This increase is particularly notable in Western countries, with obesity considered a major risk factor [11,12].

The therapeutic landscape for advanced G/GEJ adenocarcinomas is advancing with new targeted therapeutics and immunotherapies, highlighting the importance of biomarker testing to inform treatment decisions. Current international consensus guidelines from the National Comprehensive Cancer Network (NCCN), the European Society for Medical Oncology (ESMO), and Pan-Asian guidelines specify that clinical management of advanced G/GEJ adenocarcinoma requires immunohistochemistry (IHC) and/or molecular testing for Human Epidermal Growth Factor Receptor 2 (HER2/ERBB2) status, mismatch repair (MMR) or microsatellite instability (MSI) status, and Programmed Death-Ligand 1 (PD-L1) expression [13,14,15]. NCCN consensus guidelines also recommend testing for Neurotrophic Tyrosine Receptor Kinase (NTRK) fusions [13]. These recommendations align with recently published Canadian best practices for the management of patients with advanced G/GEJ adenocarcinoma and allow for the identification of patients who can benefit from anti-HER2 therapies, immune checkpoint inhibitors (ICIs), and TRK inhibitors [6]. HER2 overexpression occurs in about 12–23% of G/GEJ adenocarcinomas, MSI-high/MMR deficiency in about 8–11%, and high PD-L1 expression in 60% [13,16,17].

Access to new biomarker tests is crucial as new targeted therapies and immunotherapies come into clinical use. Claudin 18 (CLDN18) overexpression is a biomarker that is not currently in clinical use in Canada that predicts response to anti-CLDN18.2 therapy and occurs in 38–43% of patients with G/GEJ adenocarcinoma [18,19]. The Claudin 18 IHC assay detects both isoforms of CLDN18: the CLDN18.1 isoform, which is predominantly expressed in normal and neoplastic lung tissue, and CLDN18.2, which is expressed in normal gastric tissue, gastric/GEJ adenocarcinoma, and other malignancies [20,21,22,23,24,25,26,27,28]. Positive survival results for anti-CLDN18.2 therapy with zolbetuximab, a chimeric monoclonal antibody against CLDN18.2, in patients with locally advanced, unresectable, or metastatic G/GEJ adenocarcinoma with CLDN18 overexpression have been demonstrated in two phase 3 randomized clinical trials when combined with standard first-line chemotherapy [18,19]. In addition, Fibroblast Growth Factor Receptor 2b (FGFR2b) overexpression occurs in about 30% of GCs and is an emerging biomarker that may predict response to anti-FGFRb therapy. Positive results have been demonstrated in a phase 2 clinical trial, with phase 3 trials currently ongoing [29].

In Canada, most patients with advanced G/GEJ adenocarcinoma currently receive HER2, MMR and/or MSI, and PD-L1 testing to support decision-making for targeted first-line therapy. However, imminently, CLDN18 IHC testing will likely also be required as the option of anti-CLDN18.2 therapy becomes available. CLDN18 IHC is a new biomarker test that is not currently in clinical use for G/GEJ adenocarcinoma or any other cancer disease site. Therefore, a multidisciplinary pan-Canadian expert working group was convened to develop guidance for pathologists and oncologists on the implementation of CLDN18 IHC testing. The expert working group reviewed the literature on CLDN18 IHC in G/GEJ adenocarcinoma and developed consensus recommendations on the testing algorithm, as well as pre-analytical, analytical, and post-analytical considerations. The objective of the work was not to duplicate existing international guidelines on testing of HER2, MMR and/or MSI, and PD-L1, but to highlight considerations for the new CLDN18 IHC test, and also provide recommendations for predictive biomarker testing in G/GEJ adenocarcinoma that will optimize testing processes in Canada. These are the first national consensus recommendations on biomarker testing in upper gastrointestinal cancers in Canada created by a multidisciplinary working group.

## 2. Methods

### Consensus Recommendations

A multidisciplinary working group met twice to develop consensus recommendations for CLDN18 IHC and other predictive biomarker testing in G/GEJ adenocarcinoma. For the first virtual meeting, the working group consisted of eight pathologists and one medical oncologist. Subsequently, it was expanded to include one additional pathologist and two additional medical oncologists for an in-person consensus meeting on 30 October 2023, in Toronto. Members of the working group were chosen for their expertise in biomarker testing related to gastric and gastroesophageal junction (GEJ) adenocarcinoma. They are recognized as national opinion leaders in this field. Additionally, the group comprised representatives from the four major Canadian provinces to ensure that the recommendations have pan-Canadian relevance and applicability.

The first virtual meeting discussion focused on defining the scope of the group’s work and agreement on the methodology. Following this meeting, a literature search of the Medline database was performed to support the development of CLDN18 IHC testing recommendations, using keywords related to CLDN18 IHC, zolbetuximab, and G/GEJ adenocarcinoma. This search retrieved 321 unique records. After title and abstract screening to assess relevance, 94 articles were reviewed to gather evidence for recommendations for CLDN18 IHC testing in G/GEJ adenocarcinoma. Current international guidelines (NCCN, ESMO, and ASCO) were used as a starting point for recommendations regarding HER2, MMR, MSI, PD-L1, and NTRK testing, with additional targeted literature searching as needed to support the development of Canadian consensus recommendations. Recommendations were drafted by a steering committee of three pathologists and one medical oncologist.

The objective of the in-person consensus meeting was to discuss and revise the draft recommendations. Following this meeting, the revised recommendations were sent out to the working group members for review.

The revised recommendations were also reviewed by an external pan-Canadian multidisciplinary panel for input on potential implementation challenges and to ensure the recommendations were clear. The external panel was composed of 7 pathologists and 4 medical oncologists. The panel’s input was integrated into the final recommendations.

Patient advocates were also invited to review the recommendations.

Generative artificial intelligence technology was not used for any aspect of this work, including for the text, figures, tables, or any other content.

## 3. Results and Discussion

### 3.1. Predictive Biomarker Testing in Patients with Gastric or Gastroesophageal Junction Adenocarcinoma

The recommendations from the expert working group are summarized in Table 1. IHC and/or molecular testing for HER2, MMR and/or MSI, and PDL-L1 status are currently required for the clinical management of patients with locally advanced, unresectable, or metastatic G/GEJ adenocarcinoma [6]. HER2 overexpression is used to select patients for anti-HER2 therapies. MSI and/or MMR testing are used to select patients who may benefit from immune checkpoint inhibitors (ICIs) in combination with chemotherapy. PD-L1 positivity is not required for the use of ICIs; however, results for these biomarkers are useful for treating clinicians, since the benefits of ICIs are greater for patients with increasing combined positive scores (CPSs) of ≥ 1, 5, or 10 [30,31,32,33]. Although anti-CLDN18.2 monoclonal antibody therapy zolbetuximab is not yet approved by Health Canada, IHC testing for CLDN18 expression will also be required to identify patients who can benefit from this therapy.

**Figure 1:**
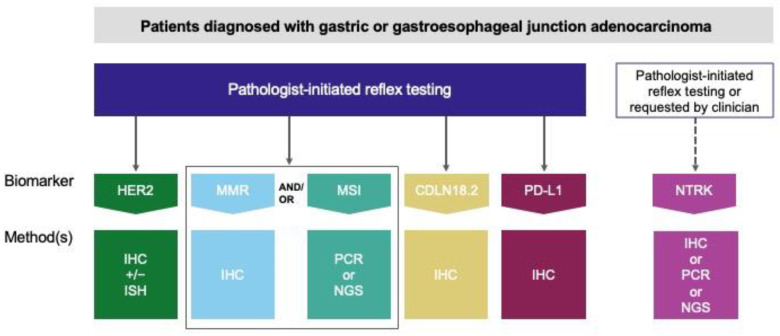
Predictive biomarker testing for patients with gastric or gastroesophageal junction adenocarcinoma.

The expert working group recommends that HER2, MMR and/or MSI, PD-L1, and CLDN18 biomarkers should be tested in all G/GEJ adenocarcinoma patients at diagnosis (Figure 1). Although results will only be immediately actionable in patients with advanced disease, testing at diagnosis ensures that test results are available when they are needed for clinical decision-making. In addition, because most patients are asymptomatic until the disease progresses to advanced stages, most patients are diagnosed at advanced stages when the results of this testing will be relevant [4]. In the setting of G/GEJ adenocarcinoma, where there are multiple first-line targeted therapies and immunotherapies available for advanced disease, it is important to have all the relevant biomarker results available up-front to the medical oncologist to support treatment decision-making [17,18,19,31,34,35].

The expert working group further recommends that testing for HER2, MMR and/or MSI, PD-L1, and CLDN18 be carried out reflexively, initiated by the pathologist at the time of diagnosis (Figure 1). Although reflex testing has not been studied in the context of G/GEJ adenocarcinoma, evidence from other disease sites, particularly lung cancer, has demonstrated that reflex testing improves biomarker testing rates and time to treatment, and is cost-effective and efficient [36,37,38,39,40]. Therefore, it is anticipated that reflex testing of predictive biomarkers in patients with G/GEJ adenocarcinoma will support the timely initiation of targeted and immunotherapy treatments.

The expert working group emphasized the importance of reflex testing for all of the recommended biomarkers: HER2, MMR/MSI, CLDN18, and PD-L1. In rare cases where limited tissue is available, testing of HER2 and MMR and/or MSI should be conducted up front, and more tissue should be obtained to test for CLDN18 and PD-L1 expression.

To preserve tissue and facilitate the timely return of results to treating clinicians, the expert working group recommends that it would be optimal to perform the testing and reporting within the same institution; however, this may not be possible in all centres, as some may not have validated in-house testing for all recommended biomarkers in G/GEJ adenocarcinomas.

NTRK fusions appear to be rare in patients with G/GEJ adenocarcinoma [41,42,43]. Therefore, depending upon the preferences and resources of the laboratory, testing for NTRK fusions could be included at the time of reflex testing for other biomarkers, or requested by the treating oncologist when third-line therapy or beyond is being considered. Patients with G/GEJ adenocarcinoma whose tumours have NTRK fusions may be considered for pan-TRK inhibitor therapy [6].

**Table 1:** Summary of predictive biomarker testing recommendations for patients with gastric or gastroesophageal junction adenocarcinoma.

Testing Algorithm
All patients with G/GEJ adenocarcinoma should be tested at diagnosis for the following panel of biomarkers: HER2, MMR deficiency/MSI, CLDN18 expression, and PD-L1 expression. Optimally, testing for all predictive biomarkers should be carried out reflexively in the same laboratory, concurrently, if possible. NTRK fusions appear rare in G/GEJ adenocarcinomas. Depending on laboratory preference, the test may be included as part of reflex testing or may be requested at the discretion of the treating oncologist when third-line therapy or beyond is being considered.
Pre-analytic Considerations
4 Testing of primary tumour tissue specimens is preferred, but testing of metastatic tumour specimens is also reasonable. Most clinical studies have used primary tumour specimens for biomarker testing, as there is reasonable concordance between biomarker results from primary and metastatic tumour specimens. Specimens used for testing should have adequate tumour cellularity, and this may guide which specimen to use for testing. Cell blocks from specimens that have been in cytology/alcohol-based fixatives should only be used if adequate validation has been performed. 5 For biopsy specimens, multiple biopsy fragments (at least six) are recommended to assess predictive biomarkers as there is known intratumoural heterogeneity for predictive biomarkers in GC. However, this does not limit testing of cases with minimal tumours present, if the minimum number of tumour cells (for example, considered to be 100 for PD-L1 testing) is present. 6 Optimally, testing should be carried out on one block that is representative of the tumour, with adequate cellularity. As noted in the guidelines from the College of American Pathologists, the American Society for Clinical Pathology, and the American Society of Clinical Oncology as well as PD-L1 testing guidelines from the Canadian Association of Pathologists, more than one block may be selected if different morphologic patterns are present, or if the minimum number of tumour cells is insufficient in one block. 7 If specimens are being sent out for molecular testing, they should be sent to the testing laboratory as quickly as possible using a courier service, with travel time preferably less than 3 days.
Analytic Considerations
8 On-slide controls with positive tissue, negative tissue, and limit of detection (system level control) tissue should be used for all IHC predictive biomarker tests. Tissues to be used as controls should align with recommendations from the individual test kits. For CLDN18 IHC, the recommended control is gastric mucosa containing intestinal metaplasia. An additional control of a positive tumour specimen may also be used. 9 Biomarker testing should be performed by a licensed, accredited laboratory and reported by pathologists trained to read the specific biomarker(s) being tested. Testing should be performed using either of the following: (a) A clinically validated commercial companion diagnostic assay following appropriate verification in accordance with the manufacturer’s requirements. (b) A laboratory-developed test that is validated in accordance with fit-for-purpose principles against a clinically validated reference standard (e.g., a companion diagnostic assay as described above). (c) Considering the multiple antibodies available for PD-L1, if the laboratory is not going to be using the standard PD-L1 pharmDx companion diagnostic kit with antibody clone 22C3 for assessment of patients for consideration of pembrolizumab therapy, then during validation of any laboratory-developed PD-L1 test, results must be compared to results from the companion diagnostic kit [44]. If the laboratory is planning on validating only one PD-L1 antibody (either 28-8 or 22C3) when results from both might be needed depending on the treatment being considered, the laboratory should validate the antibody in use against the other antibody, which may require support from a laboratory that has the well-validated alternative antibody.
Post-analytic Considerations
10 Biomarker test results should be reported within 10 working days of test requisition, with all biomarker test results compiled by the pathologist and listed sequentially in one pathology report. Synoptic reporting should be carried out wherever possible. An addendum with the compiled biomarker results should be added to the original diagnostic report when predictive biomarker testing is completed.

### 3.2. Pre-Analytic Considerations

The accurate diagnosis and characterization of biomarkers in G/GEJ adenocarcinoma relies on the quality of specimens obtained for testing. Pre-analytical factors, which encompass all processes from sample collection to processing, can significantly impact the reliability and validity of test results [45]. Where available, laboratories should follow existing guidelines for testing of biomarkers that are currently used in G/GEJ adenocarcinoma—HER2, MMR/MSI, PD-L1, and NTRK [44,46,47,48]. As CLDN18 testing is not yet in clinical use in North America or Europe, there are no published guidelines yet that provide recommendations on pre-analytical considerations for CLDN18 IHC. After reviewing the literature, the expert working group developed several recommendations that apply to CLDN18 IHC as well as other biomarkers in G/GEJ adenocarcinoma. These recommendations align with published guideline recommendations for HER2, MMR/MSI, PD-L1, and NTRK biomarker testing [44,46,47,48].

The expert working group recommends that biomarker testing be performed on primary tumour specimens since this is typically the specimen type used to test biomarkers in clinical trials for anti-HER2-targeted therapies, ICIs, and anti-CLDN18.2 therapy in G/GEJ adenocarcinoma [18,19,31,49,50,51]. However, testing of metastatic tumour specimens can be undertaken, especially when primary tumour tissue is not available or when the metastatic site is more accessible for biopsy. Although G/GEJ adenocarcinoma biomarkers exhibit intratumoural heterogeneity, there is a reasonable concordance of at least 80 to 90% between biomarker results from primary and metastatic specimens. For example, between matched primary and metastatic GC specimens, the concordance for HER2 expression status by IHC is 93 to 95% [52,53,54]. For CLDN18 IHC, in one study, 7% of tumour specimens had strong heterogeneity, defined as tumours that had both strong (3+) and negative staining detectable, making up 50% of the tumour tissue combined [55]. Studies of matched primary and metastatic lymph node tumour specimens found 82 to 86% concordance for CLDN18 status, and concordance of greater than 70% for matched primary and distant metastatic specimens, and matched specimens before and after chemotherapy [21,23,55,56]. The concordance of PD-L1 CPS ≥ 1 varies between studies, but can be above 90%, and is higher for matched pairs in which the primary specimen is negative [57,58,59,60].

While specific information on studies involving cytological G/GEJ adenocarcinoma specimens is scarce, a growing body of research in other solid tumours indicates that biomarker testing on cytological samples may be feasible provided that pre-analytical conditions, such as accurate specimen collection and handling, appropriate triage of the samples, and processing, are met [61,62]. Cytology fixatives contain alcohol, which can alter epitope presentation on cells and, thus, immunohistochemistry results; specific validation is needed to ensure that the test works under these conditions. Guidelines for HER2 biomarker testing in gastroesophageal adenocarcinoma allow for the use of cytology specimens from cell blocks, although this is not preferred; a low threshold for performing in situ hybridization is likely to be of use [47]. PD-L1 IHC guidelines have addressed the use of cytology specimens [44]. The use of cytology specimens for CLDN18 IHC has not been well studied, although some studies have found that CLDN18 IHC can be performed successfully on cytology specimens [63,64]. The expert working group recommends that specimens that have been in cytology/alcohol-based fixatives should only be used if adequate test validation has been performed.

Due to the intratumoural heterogeneity of predictive biomarkers in G/GEJ adenocarcinoma, the expert working group recommends that at least six biopsy fragments should be used to assess predictive biomarkers. However, specimens with minimal tumour present may still be tested, as long as the minimum number of tumour cells for each biomarker being tested is present, as defined by the manufacturer’s instructions for the companion diagnostic (CDx) assay.

The expert working group recommends that molecular biomarker testing be performed on a single representative block with adequate tumour cellularity. This is ideal for several reasons, including consistency, resource efficiency, and specimen preservation as well as ease of interpretation. However, guidelines for HER2 biomarker assessment in G/GEJ adenocarcinoma also allow for testing of multiple blocks when different morphological profiles or tumour heterogeneity is anticipated [47]. In addition, guidelines for PD-L1 testing also recommend that multiple blocks should be used if the minimum number of tumour cells can be reached by combining blocks [44]. In line with this recommendation, the expert working group also recommends that for testing of biomarkers in G/GEJ adenocarcinoma, more than one block can be selected if different morphologic profiles are present. A minimum of 50 viable tumour cells is required for CLDN18 and MMR IHC assays, while 100 viable cells are required for PD-L1 IHC [28,44,65,66].

To ensure that the treating oncologist has access to biomarker results in a timely fashion, the expert working group recommends that specimens sent to another laboratory for testing be sent using a courier service, with travel time ideally less than three days. Specimen reception and time to IHC testing should also be minimized.

### 3.3. Analytic Considerations

**Figure 2:**
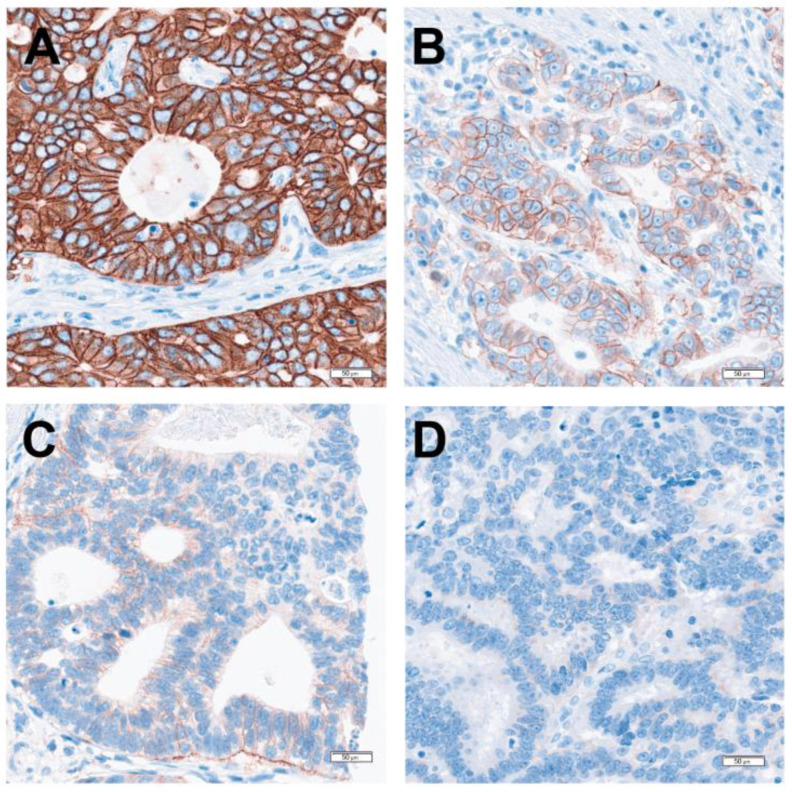
HER2 IHC (clone 4B5; performed with Ventana BenchMark Ultra according to the manufacturer’s protocol) showing scoring of representative gastric adenocarcinoma cases. (**A**) Positive 3+ staining demonstrating strong complete, basolateral, and lateral membranous reactivity in the tumour cells. (**B**) Positive 2+ staining demonstrating weak to moderate complete, basolateral, and lateral membranous reactivity in the tumour cells. (**C**) Negative 1+ staining demonstrating faint/barely perceptible membranous reactivity in the tumour cells. (**D**) Negative 0 staining demonstrating no reactivity or membranous reactivity in any tumour cell.

**Figure 3:**
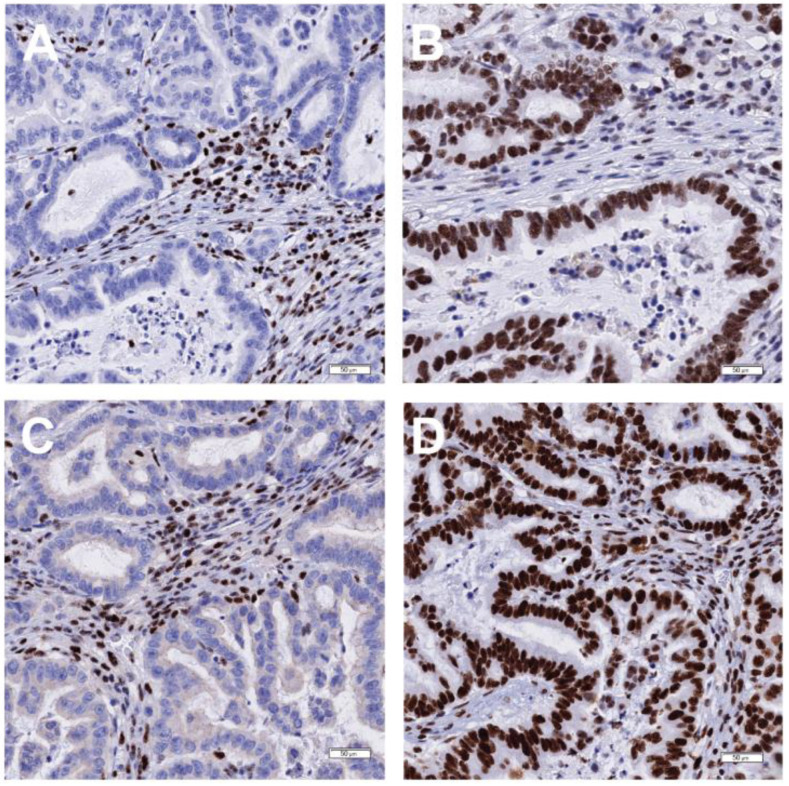
MMR IHC (performed with Ventana BenchMark Ultra according to the manufacturer’s protocol) with representative gastric adenocarcinoma cases showing the most common pattern of MMR deficiency. (**A**) MLH1(M1) showing loss of nuclear expression in the cancer cells but retention in surrounding inflammatory cells. (**B**) PMS2 (clone A16-4) showing loss of nuclear expression in the cancer cells but retention in surrounding inflammatory cells. (**C**) MSH2 (clone G219-1129) showing retained expression in the tumour cells. (**D**) MSH6(SP93) showing retained expression in the tumour cells.

**Figure 4:**
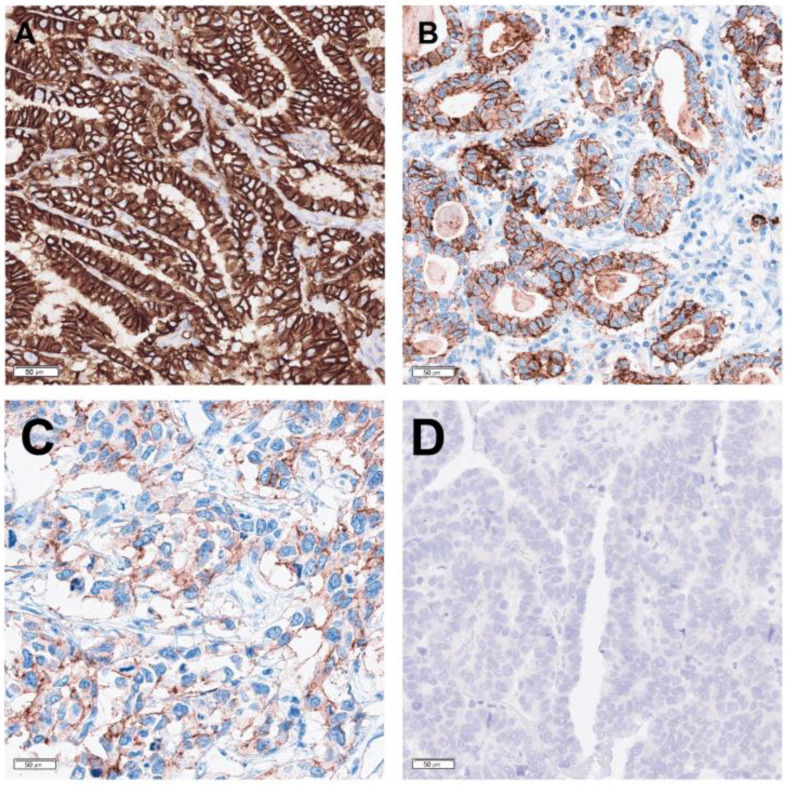
CLDN18 IHC (clone 43-14A; performed with Ventana BenchMark Ultra according to the manufacturer’s protocol) showing scoring of representative gastric adenocarcinoma cases. (**A**) Positive 3+ staining demonstrating strong membranous reactivity. (**B**) Positive 2+ staining demonstrating moderate membranous reactivity. (**C**) Negative 1+ staining demonstrating weak membranous reactivity. (**D**) Negative 0 staining demonstrating no membranous reactivity.

**Figure 5:**
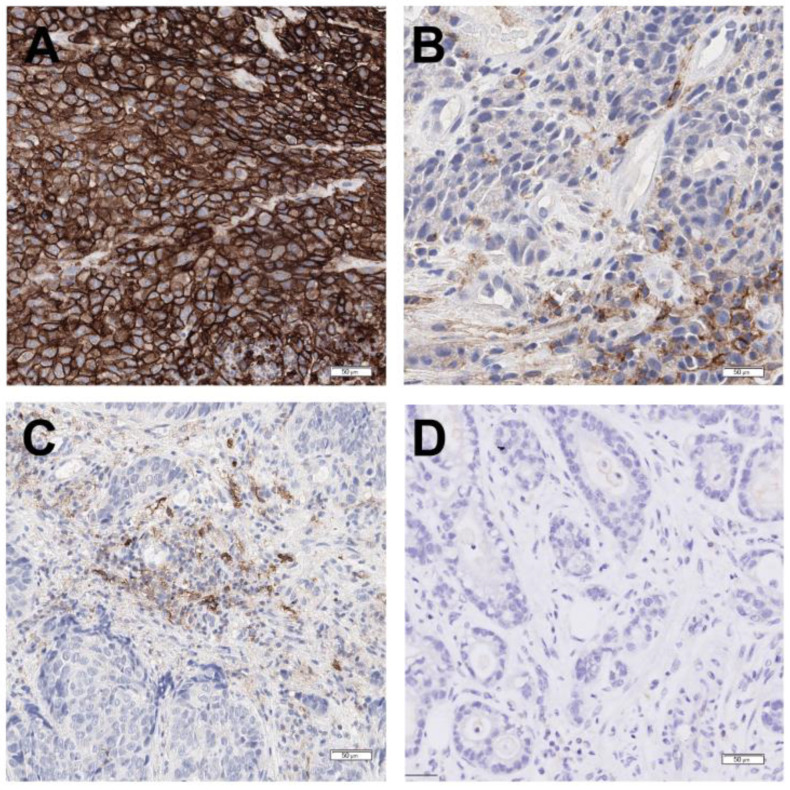
PD-L1 IHC (PD-L1 IHC 28-8 pharmDx from Agilent; performed on the Autostainer Link 48 according to the manufacturer’s protocol) scoring of representative gastric adenocarcinoma cases. Scoring is performed using the combined positive score (CPS), a ratio of PD-L1-staining cells (tumour cells, lymphocytes, and macrophages) relative to viable tumour cells. (**A**) High CPS showing high tumour cell staining (CPS on the whole slide was 100). (**B**) Low CPS with focal tumour and lymphocyte and macrophage staining (CPS on the whole slide was 5). (**C**) Low CPS with lymphocyte and macrophage staining but without tumour cell staining (CPS on the whole slide was <1). The positive lymphocytes were very focal compared to the tumour. (**D**) CPS of 0.

Currently, immunohistochemistry (IHC) is the predominant method used to test predictive biomarkers in G/GEJ adenocarcinoma due to the specific biomarkers being tested (Figure 1). Images of HER2, MMR, CLDN18, and PD-L1 IHC assays of representative cases are shown in Figure 2, Figure 3, Figure 4 and Figure 5, respectively. All biomarkers recommended by the expert working group for reflex testing in patients with G/GEJ adenocarcinoma—HER2, MMR, PD-L1, and CLDN18—can be evaluated using IHC [13,18,19,43]. Molecular methods are sometimes a feasible alternative, depending on the biomarker, or may be used as a confirmatory or secondary method following initial IHC testing. For example, IHC is recommended for initial assessment of HER2 overexpression, followed by fluorescent in situ hybridization (FISH) or other in situ hybridization (ISH) methods for specimens with equivocal results from IHC [41,47]. Although MMR deficiency is most often assessed with IHC, which is available in most laboratories, some laboratories may have polymerase chain reaction (PCR) or next-generation sequencing (NGS) tests for MSI, which is a reasonable alternative, as microsatellite instability results from MMR deficiency [67]. One study of a large number of GC specimens demonstrated over 98% concordance for results from MMR IHC and PCR-based MSI testing [68]. If NTRK testing is undertaken, there is some evidence that pan-TRK IHC can be an effective screening method in GC specimens, but fusions must be confirmed via molecular testing [69]. PCR or NGS may also be used to detect NTRK fusions [70]. The risk of tissue depletion should be considered when ordering molecular testing for NTRK fusions or MSI testing by PCR or NGS.

It is important to note that although different scoring methods have been used to interpret PD-L1 immunostaining in tumour specimens from different cancers, the expert working group supports the use of the combined positive score (CPS) as the standard for assessing PD-L1 expression in G/GEJ adenocarcinoma specimens (Figure 1). CPS has typically been used in phase 3 clinical trials of ICIs in GC, and it is more predictive of ICI benefit than the tumour proportion score (TPS) [31,33,51,71,72]. Tumour Area Positivity (TAP) scoring has also been published but is not recommended [73].

The scoring algorithm for CLDN18 IHC in gastric/GEJ carcinoma defines the cutoff for positivity as greater than or equal to 75% of viable tumour cells demonstrating moderate to strong membranous CLDN18 staining (2+ or 3+) [28]. Membranous staining may be complete, basolateral, or lateral [65]. Furthermore, the HER2 “magnification rule” can be used to assess the intensity of staining, with 3+ intensity corresponding to strong brown immunoreactivity with a chicken wire appearance, visible at 50× magnification. Staining of 1+ or 2+ intensity requires higher magnification: 400× for 1+ staining, and 100–200× for 2+ staining [65,74]. The selection of appropriate controls is critical for the accurate interpretation of IHC testing. Following best practices for the use of positive and negative controls for diagnostic IHC [75,76], the expert working group recommends that on-slide controls with positive (high expressing) tissue, positive (low limit of detection) tissue, and negative tissue should be used for predictive biomarker IHC testing in G/GEJ adenocarcinoma. When using commercial companion diagnostic (CDx) kits, tissues to be used as controls should align with recommendations in the instructions for use from the individual test kits. For tests where the laboratory does not use a commercial CDx kit or there is no commercial kit available, the recommendation above should be followed to select appropriate control tissues. For example, for CLDN18 IHC, the expert working group recommends the use of gastric mucosa containing intestinal metaplasia as a control tissue. This tissue displays strong positive CLDN18 staining in normal gastric epithelial cells [23], weak to moderate (limit of detection) staining in epithelial cells in areas of metaplasia [77,78], and absence of staining in lamina propria, lymphocytes, smooth muscle, blood vessels, and peripheral nerve [43], which can be used as the negative control.

As with any biomarker testing, predictive biomarker testing in G/GEJ adenocarcinoma may be performed using commercial CDx assays or laboratory-developed tests (LDTs). Laboratories should perform appropriate verification studies for commercial CDx assays, in accordance with the manufacturer’s requirements. LDTs should be validated in accordance with fit-for-purpose principles against a clinically validated reference standard [79,80]. A clinically validated reference standard, for the purposes of biomarker-driven patient selection, means a specific diagnostic assay that was used to assess specimens for patients with a specific disease to select those who respond to a specific drug in a clinical trial [44]. LDTs have commonly been used in Canada for assessment of PD-L1 expression; however, there are also two different CDx assays. The 28-8 pharmDx assay (Dako North America, Inc., Carpinteria, CA, USA), which uses the 28-8 antibody clone, may be used as an aid in the assessment of patients for whom nivolumab treatment is being considered, while the 22C3 pharmDx assay (Dako North America, Inc.) is used to assess PD-L1 expression in patients for whom pembrolizumab is being considered. Thus, if a laboratory intends to use the 28-8 antibody clone to assess PD-L1 expression in G/GEJ adenocarcinoma tumour specimens for consideration of pembrolizumab therapy, results from the LDT must be validated against results from the 22C3 pharmDx commercial CDx assay for any specific readout and cutoff point. In addition, an LDT using the 28-8 antibody clone must also be validated against the 28-8 pharmDx commercial assay. Similarly, both pharmDx assays are cleared for use with the Autostainer Link 48 staining platform (Agilent Technologies), and use of any other staining platform turns the assay into an LDT that also has to be validated using the above principles and cleared platform. Currently funded ICI therapies in Canada use the 28-8 and 22C3 antibody clones, and if additional ICI therapies come into use that use different antibodies, this would also need to be taken into consideration for assay validation.

Predictive biomarker testing for G/GEJ adenocarcinoma should be performed by licensed laboratories and reported by pathologists who have proficiency in reading out each of the specific biomarkers being tested. Pathologist training is important to ensure accuracy and consistency in the reporting of biomarker results [79].

### 3.4. Post-Analytic Considerations

The biomarker and molecular pathology reports provide critical information for the medical oncologist to support treatment decision-making. Discussions at multidisciplinary tumour boards are essential in selecting the optimal pathway for patient treatment, considering the complexity of the predictive biomarker results. It is mandatory for the biomarker testing results to be reported quickly so that treatment can be initiated in a timely fashion. The expert working group recommends that biomarker test results should be reported within ten working days of test requisition. This provides results within a clinically useful timeframe and aligns with HER2 biomarker testing guidelines [47]. Biomarker results should be compiled by the pathologist and listed sequentially in one report, allowing the medical oncologist to find all the results needed to make treatment decisions in one place. In addition, the report should be structured in a clear and concise format for ease of interpretation. If testing is carried out externally and/or piecemeal, then an addendum with the compiled biomarker results should be added to the original diagnostic report and flagged to the oncologist, if possible, once all biomarker testing is completed.

The expert working group recommends that synoptic reporting should be carried out wherever possible. Synoptic reporting has been shown to enhance the quality of surgical pathology reports for GC, improving the completeness of information in the report [81].

### 3.5. Challenges for the Implementation of Reflex Predictive Biomarker Testing for HER2, MMR, PD-L1, and CLDN18 in Canada

An external pan-Canadian, multidisciplinary panel of oncologists and pathologists provided input on potential challenges for implementing predictive testing for HER2, MMR, PD-L1, and CLDN18 as reflex tests at diagnosis of G/GEJ adenocarcinoma. The most commonly mentioned barrier to implementation was the lack of sufficient human resources. There are not enough trained laboratory personnel and pathologists to accommodate the growing number and increasing complexity of predictive biomarkers required for the clinical management of oncology patients. Increased operational costs for performing increasing numbers of biomarker tests is an additional challenge. For institutions that are not able to support in-house testing for these biomarkers due to a lack of sufficient resources, the need to send samples out for testing creates a barrier to testing and may increase the turnaround time for results. The costs of validation for LDTs when new tests are introduced to the laboratory also need to be considered. The changing biomarker landscape requires ongoing educational efforts for gastroenterologists, surgeons, oncologists, and pathologists. There is a need for ongoing multidisciplinary discussions between specialists involved with caring for these patients, as well as health system policymakers, to develop solutions that will address these barriers.

### 3.6. Patient Perspective on These Recommendations

Patient advocates reviewed the recommendations from the expert working group and provided perspectives on their impact on patients. They commented that the recommendations for reflex testing of HER2, MMR and/or MSI, PD-L1, and CLDN18 at diagnosis are critical for treatment decision-making and for patients to be able to access targeted therapies and immunotherapies. The patient advocates noted that it is important for treating physicians to discuss the results of biomarker testing with their patients, both when results are positive and when they are negative, and to explain the implications of the results for the patient’s treatment plans. They emphasized the importance of incorporating new biomarkers into the testing algorithm as new therapies become available.

## 4. Conclusions and Future Directions

Predictive biomarker testing results are critical for decisions about treatment options in patients with locally advanced, unresectable, or metastatic G/GEJ adenocarcinoma. The expert working group recommends reflex testing for HER2, MMR and/or MSI, CLDN18, and PD-L1 in all patients with G/GEJ adenocarcinoma at the time of diagnosis. Testing for NTRK fusions may be included as a reflex test or requested by the treating clinician when third-line therapy is being considered. Additional recommendations herein highlight important pre-analytic, analytic, and post-analytic test considerations for predictive biomarker testing in G/GEJ adenocarcinoma. These evidence-based recommendations from a group of experts provide valuable information for laboratories providing biomarker testing in gastric/GEJ adenocarcinoma and for medical oncologists managing these patients. As CLDN18 IHC comes into use, recommendations in clinical practice guidelines from national and international societies will likely provide further guidance for pathologists and oncologists. Additionally, in the future, by integrating artificial intelligence and machine learning algorithms, digital pathology platforms have the potential to improve diagnostic accuracy and efficiency in predictive biomarker testing. Other future considerations include the changing biomarker landscape as new therapies emerge, which will likely require additional biomarker tests: these recommendations will need to evolve to support those tests as well.
